# LDLK-U-Mamba: An Efficient and Highly Accurate Method for 3D Rock Pore Segmentation

**DOI:** 10.3390/s25227039

**Published:** 2025-11-18

**Authors:** Guojun Chen, Huihui Li, Chang Liu, Pengxia Li, Yunyi Kong

**Affiliations:** 1 Qingdao Institute of Software, College of Computer Science and Technology, China University of Petroleum (East China), Qingdao 266580, China; chengj@upc.edu.cn (G.C.); z23070142@s.upc.edu.cn (C.L.); s23070018@s.upc.edu.cn (P.L.); s24070043@s.upc.edu.cn (Y.K.); 2 Shandong Key Laboratory of Intelligent Oil & Gas Industrial Software, Qingdao 266580, China

**Keywords:** 3D rock pore segmentation, Mamba, lightweight

## Abstract

Three-dimensional rock pore segmentation is crucial in fields such as geology and petroleum exploration, holding significant importance for oil and gas resource exploration and development. However, existing segmentation methods still present two main limitations: (1) they fail to capture the spatial relationships of pores in 3D when directly applied to 3D rock pore segmentation, inevitably leading to inaccurate segmentation results; (2) they struggle to apply efficiently in resource-constrained scenarios due to the high computational complexity and costly computational demands. To solve the above issues, we propose a novel and lightweight method based on the Mamba architecture, termed LDLK-U-Mamba, for precise and efficient 3D rock pore segmentation. Specifically, we design a Lightweight Dynamic Large Kernel (LDLK) module to capture global contextual information and develop an InceptionDSConv3d module for multi-scale feature fusion and refinement, further yielding more accurate segmentation results. In addition, the Basic Residual Depthwise Separable Block (BasicResDWSBlock) module is proposed to utilize depthwise separable convolutions and the Squeeze-and-Excitation (SE) module to reduce model parameters and computational complexity. Extensive qualitative and quantitative experiments demonstrate that our LDLK-U-Mamba outperforms current mainstream segmentation approaches, validating its effectiveness for rock pore segmentation—particularly in capturing the 3D spatial relationships of pores.

## 1. Introduction

In geological science and geology research, the analysis of rock structures and dimensional structures is crucial for understanding the evolutionary processes of underground oil and gas reservoirs [1]. In particular, the pore structures within rock images play a vital role in determining water resource planning, geothermal energy utilization and underground resource exploration [2]. However, due to the inherent complexity of rock structures and the inability to clearly separate pore boundaries from the rock matrix, segmenting pore regions within rock images presents a formidable challenge.

In the task of segmenting two-dimensional images, such as in the fields of geology [3], remote sensing [4], and medicine [5], the most commonly used deep learning architecture is U-Net [6]. Beyond U-Net, existing research has proposed numerous improved network architectures, such as Attention U-Net [7], U-ResNet [8], Dense U-Net [9], SFMRNet [10] and TransUNet [11]. However, these 2D segmentation methods rely solely on feature extraction from a single planar image, resulting in the loss of depth information and spatial correlations within rock pores. This leads to discrepancies between segmentation results and actual pore morphology. To address these limitations, 3D segmentation methods have gradually emerged as a key research focus.

In recent years, 3D U-Net [2] and 3D U-ResNet [8] have been applied to mineral segmentation in 3D rock images. However, they struggle to capture edge textures, resulting in low segmentation accuracy. Given the limited number of existing networks for 3D rock image segmentation, while popular networks in 3D medical image segmentation—such as 3D SegNet [12], Kiu-net [13], nnUNet [14], V-Net [15], UNETR [16] and U-Mamba [17]—demonstrate remarkable performance in medical image segmentation, but they struggle to segment elongated pores effectively when applied to rock segmentation.

In our study, we propose a segmentation-based LDLK-U-Mamba model capable of segmenting pores in 3D rock images efficiently and accurately. Specifically, we propose a lightweight 3D rock image pore segmentation method based on the Mamba architecture, termed LDLK-U-Mamba, for segmenting pores in 3D rock images. We propose using an LDLK module during the encoding phase to capture global contextual information and utilizing the InceptionDSConv3d module during the decoding phase for multi-scale feature fusion and refinement, achieving more accurate segmentation results. Additionally, we propose the BasicResDWSBlock module, utilizing deep separable convolutions and the SE module to reduce model parameters and computational complexity. We conduct several comparative experiments for rock pore segmentation to illustrate performance differences among algorithms. Experimental results on the Leopard Sandstone Images dataset demonstrate that our model achieves 99.38% accuracy, 99.62% Dice, and 99.25% IOU, while reducing parameters from 42.12 M to 13.97 M, and computational FLOPs decreased from 973.91 GFLOPs to 426.64 GFLOPs. Compared with other 3D segmentation networks, our proposed LDLK-U-Mamba demonstrates outstanding comprehensive performance.

Overall, the contributions of our research can be summarized as follows:

(1) We propose a 3D rock pore segmentation model, termed LDLK-U-Mamba, which is based on the mamba network for precise and efficient 3D rock pore segmentation.
(2) We propose a Lightweight Dynamic Large Kernel (LDLK) module to capture global contextual information and design an InceptionDSConv3d module to fuse and refine multi-scale features, thereby achieving more accurate segmentation results.
(3) We propose a Basic Residual Depthwise Separable Block (BasicResDWSBlock) module, which employs separable convolutions and the Squeeze-and-Excitation (SE) module to reduce model parameters and computational complexity.
(4) The comparative experiments demonstrate LDLK-U-Mamba outperforms the existing 3D segmentation networks.

## 2. Related Work

### 2.1. 2D Image Segmentation

In 2D images, accurately segmenting digital rocks into categories such as pores and rocks remains a formidable challenge [18]. Traditional image segmentation techniques, including thresholding (such as the maximum interclass variance method [19]), watershed segmentation [20], clustering analysis, and edge detection [21], rely heavily on parameter selection influenced by user experience and subjective judgment, which lead to inconsistent segmentation results [22]. Among deep learning architectures, U-Net [6] fuses multi-scale information, yet its skip connections naively concatenate features across scales without accounting for 3D structural coherence such as voxel spatial relationships and pore dimensional connectivity, thus struggling to capture fine 3D spatial details like intricate rock pore structures. Attention U-Net [7] introduces an attention mechanism in the decoder, enabling the network to focus on more important feature regions. However, it lacks the targeted modeling of geometric features, such as spatial orientation and hierarchy in 3D space, with hierarchy referring to the micro-to-macro progression of pore-related features (throats, pore clusters, and pore zones) that are interconnected in the 3D space. U-ResNet [8] introduces the residual structure from ResNet, mitigating the gradient vanishing problem in deep networks through shortcut connections. Nevertheless, it struggles to accurately distinguish subtle structural differences within the 3D space. Dense U-Net [9] enhances feature reuse efficiency, but its dense connections cause feature map counts to increase exponentially with network depth and fail to effectively capture cross-dimensional feature correlations in the 3D space. SFMRNet [10] excels in segmentation tasks for ambiguous regions in remote sensing images through its multi-feature correlation module, but it is limited to two-dimensional data processing. TransUNet [11] combines the global modeling advantages of Transformers with UNet’s capability for fine-grained detail recovery, demonstrating exceptional performance in capturing long-range dependencies. However, its reliance on two-dimensional slice processing prevents it from leveraging the spatial correlation information inherent in three-dimensional data. Zunair et al. [23] proposed Masked Supervised Learning (MaskSup) for semantic segmentation, a single-stage method that uses a Siamese network and random masking to model both short- and long-range context, addressing small target segmentation, ambiguous boundaries, and class imbalance. Like the other 2D methods above, it fails to capture fine 3D pore details. In contrast, within our proposed LDLK-U-Mamba model, the LDLK module and InceptionDSConv3d module effectively address the limitations of 2D segmentation methods in capturing 3D spatial features.

### 2.2. 3D Image Segmentation

In recent years, the advent of high-resolution micro-computed tomography (µCT) imaging of rock samples [8] has enabled the acquisition of 3D image data, paving the way for refined segmentation of rock images. In mineral segmentation within 3D rock images, while 3D U-Net [6] can capture richer contextual information spatially, it struggles to adequately capture edge and texture details, resulting in lower segmentation accuracy. Furthermore, while 3D U-ResNet [24] demonstrates improved robustness in handling complex 3D structures through residual connections, it fails to resolve the boundary distinction between pores and matrix in rock samples. In domains like medical image segmentation, 3D SegNet [12] employs a symmetric encoder–decoder architecture to optimize information recovery during upscaling. However, this approach significantly increases computational demands when processing ultra-large volumetric data. Three-dimensional KiUNet [13] proposes block-based segmentation methods, but the loss of spatial correlation between blocks often leads to edge discontinuities after stitching. Meanwhile, nnUNet [14], as a highly adaptive framework, requires multi-round hyperparameter optimization validation, inevitably extending training cycles. V-Net [15] excels at capturing three-dimensional spatial features in 3D medical images with high segmentation accuracy, but it has numerous model parameters and demands substantial computational resources. UNETR [16] combines Transformer with 3D U-Net, offering strong global modeling capabilities, yet it suffers from insufficient small-object segmentation accuracy and requires large amounts of data for training. Although the U-Mamba [17] network performs well on large-scale data due to its multi-scale feature extraction, the computational complexity of 3D convolutions still results in lengthy processing times when handling 3D data. Furthermore, when dealing with extremely fine pores in rocks, multi-scale feature extraction struggles to fully capture the details of microscopic structures, leaving room for improvement in segmentation accuracy. To date, all existing 2D or 3D rock image segmentation networks have been developed for studying rock minerals. Our work represents the first research focused on pore segmentation in 3D rock images, which is performed precisely.

### 2.3. Neural Network Lightweighting

In deep learning image segmentation and feature extraction tasks, enhancing network performance under limited computational resources and memory constraints has become a significant research focus. To address this challenge, Szegedy et al. [25] introduced the Inception module, the first network to efficiently model image features through the parallel computation of multi-scale features. Subsequently, Szegedy et al. [26] further proposed Inception-v2 and Inception-v3, introducing factorized convolutions and batch normalization to further reduce memory consumption and computational costs. To minimize computational resource requirements, Chollet et al. [27] proposed the Xception network, which further optimized the computation of convolutional operations through deep separable convolutions. Inspired by Xception, MobileNet [28] introduced a lightweight network based on deep separable convolutions, enabling efficient image classification and segmentation on mobile and embedded devices. Building upon this foundation, MobileNetV2 [29] further introduced reverse residual structures and linear bottlenecks to reduce memory requirements and computational costs. Zhao et al. [30] also proposed a lightweight 3D segmentation network based on Inception and deep separable convolutions. This approach achieved excellent segmentation results on multiple 3D medical image datasets while maintaining low computational costs, offering new insights for pore segmentation in 3D rock images.

## 3. Methods

### 3.1. Overview

**Figure 1:**
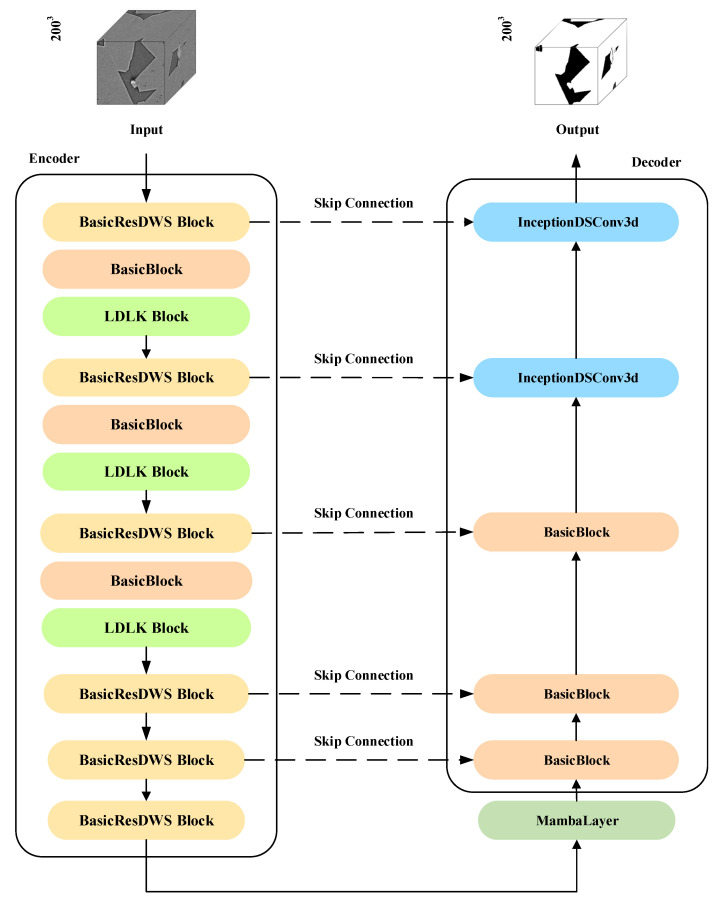
The structure of our LDLK-U-Mamba. The model includes an encoder, a decoder, and skip connections. The LDLK module in the encoder captures global contextual information, while the InceptionDSConv3d module in the decoder achieves more accurate segmentation results through multi-scale feature fusion and refinement. The BasicResDWSBlock module employs separable convolutions and the SE module to significantly reduce the model’s parameter count and computational complexity. The BasicBlock acts as a basic feature extraction unit with stacked 3D convolutions, instance normalization, and activation for 3D feature capture.

We propose a lightweight 3D rock image pore segmentation method based on the Mamba architecture, termed LDLK-U-Mamba, for segmenting pores in 3D rock images. It aims to enhance segmentation performance while reducing the number of parameters and FLOPs compared to Mamba. The structure of our LDLK-U-Mamba model is shown in Figure 1. The model includes an encoder, a decoder, and skip connections. The LDLK module in the encoder captures global contextual information, while the InceptionDSConv3d module in the decoder achieves more accurate segmentation results through multi-scale feature fusion and refinement. The BasicResDWSBlock module employs separable convolutions and the SE module to significantly reduce the model’s parameter count and computational complexity.

### 3.2. The LDLK Module

**Figure 2:**
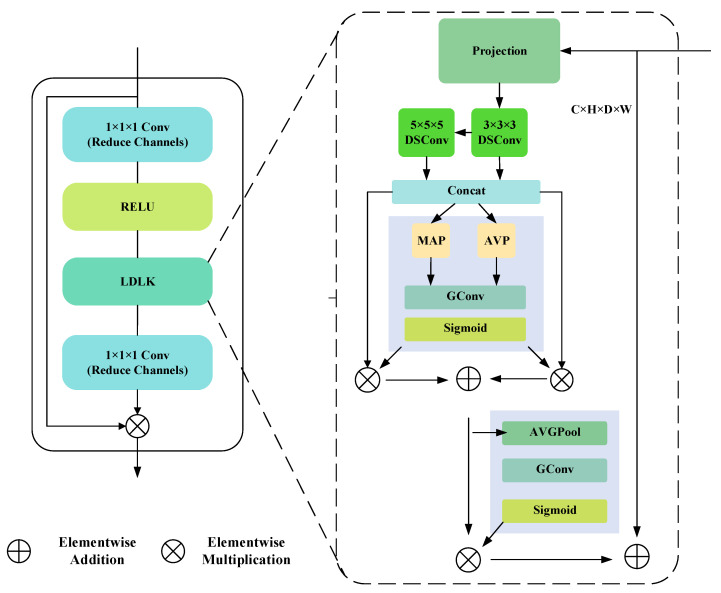
The LDLK module. This module includes Reduce Channels, a RELU, and an LDLK. Reduce Channels and ReLU effectively reduce computational load, while LDLK’s dynamic group convolution enables dynamic feature fusion with low computational overhead, efficiently utilizing global contextual information.

Molina et al. [3] achieved a refined analysis of geological segmentation through multi-source geoscience data in their study of Chile’s giant thrust belt. Similarly, in the field of rock pore segmentation, the integrated extraction of multidimensional features such as pore morphology and connectivity has become a key direction for enhancing segmentation accuracy. In existing research, Transformer models have achieved remarkable success in image segmentation due to their extensive receptive fields and ability to effectively utilize long-range contextual information across the entire image [31]. Convolutional neural networks (CNNs) can also achieve large receptive fields by employing large-sized convolutional kernels, enabling them to attain competitive performance with fewer model parameters. However, in 3D rock image segmentation, fixed-size convolutional kernels lack the flexibility to handle pores and fractures at different scales. CNNs using large convolutional kernels remain constrained in capturing multi-scale features of pore structures with significantly varying shapes and sizes. This limitation may cause models to underperform when handling complex and variable rock microstructures, particularly when precise identification of pore features of varying sizes and morphologies is required. Additionally, they fail to efficiently utilize global contextual information. To address these limitations, we propose the LDLK module with dynamic feature fusion capabilities [32]. The structure of the LDLK module is shown in Figure 2. To maintain low computational and parameter complexity, our LDLK utilizes convolutional kernel sizes (3 × 3 × 3 and 5 × 5 × 5) that are more suitable for our dataset, combined with a reasonable dilation factor (dilation = 2).

As indicated by Equation (1), let $X_{ch}^{l}$ be the input feature map, where X is the z-dimension (slice) of the sample object, *l* is the number of layers the feature map is located in, *c* is the channel dimension, and *h* is the spatial dimension information. To create the attention weights, we utilize grouped convolution (groups = 2) in conjunction with a tiny convolution kernel (3 × 3 × 3), following Zhang et al. [33]’s design of dynamic grouped convolution.

(1) $$w_{ch}=\mathrm{Sigmoid}\left(\mathrm{GConv}\left(\mathrm{AVGPool}\left(X_{ch}^{l}\right)\right)\right).$$

Second, as shown in Equation (2), we refer to the channel compression method proposed by Han et al. in [34] and propose to use channel dimensionality reduction. Let the number of channels of the input feature map be $C_{in}$, and reduce the number of channels to 1/32 of the original one by 1 × 1 × 1 convolution, then the number of channels after dimensionality reduction $C_{reduced}=\left\lfloor \frac{C_{in}}{32}\right\rfloor$,

(2) $$X_{reduced}^{l}={\mathrm{Conv}}_{1\times 1\times 1}\left(X^{l},C_{reduced}\right),$$

where $X^{l}$ ($X^{l}$ and the previously mentioned $X_{ch}^{l}$ represent a reasonable distinction in symbols to accommodate different operational requirements) is the input feature map, ${\mathrm{Conv}}_{1\times 1\times 1}$ denotes the 1 × 1 × 1 convolution operation, and $X_{reduced}^{l}$ is the feature map after dimensionality reduction. The original number of channels is then restored after activation and spatial gating unit processing, and this bottleneck structure significantly reduces the intermediate computational cost.

The above process can be expressed as Equation (3),

(3) $$X^{l}=\mathrm{LDLK}\left(\mathrm{ReLU}\left(X^{l}\right)\right).$$

In order to increase operating efficiency, we decide to utilize the lightweight ReLU function for the activation function. Overall, our design focuses more on efficient computation on the basis of guaranteeing the segmentation effect, which is suitable for the case of limited computational resources.

### 3.3. The InceptionDSConv3d Module

The task of the decoding stage is to progressively restore segmented spatial details, necessitating multidirectional and multi-scale reconstruction of the high-dimensional features extracted by the encoder. To achieve fusion and refinement of the multi-scale features, we combine the Inception module with deep separable convolutions in the decoder, proposing the InceptionDSConv3d module. The Inception module was first introduced by Szegedy et al. [25] to capture features at different scales through parallel operations of multi-scale convolutional kernels. Subsequently, Chollet et al. [27] introduced Deep Separable Convolution in 2017. This method decomposes standard convolutions into depthwise convolutions and pointwise convolutions, thereby reducing computational complexity and enhancing model performance. Therefore, we propose combining the multi-scale feature extraction of the Inception module with the efficient computation of Deep Separable Convolution to achieve the fusion and refinement of multi-scale features [35].

**Figure 3:**
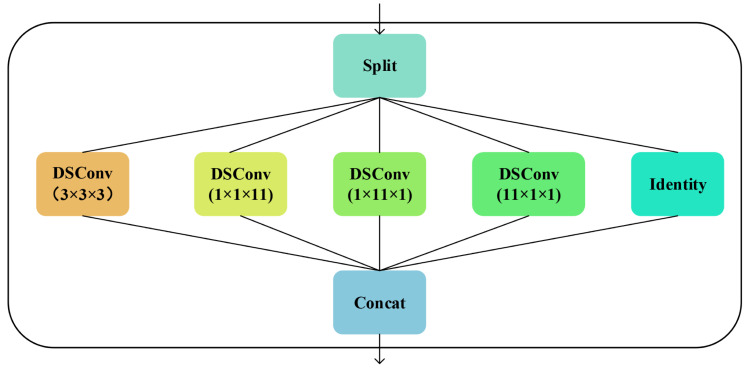
The InceptionDSConv3d module. This module divides the input channel dimensions into four depthwise separable convolutional branches and one identity branch, with the outputs of each branch ultimately concatenated. This multi-branch architecture, combined with depthwise separable convolutional kernels of varying sizes, enables fine-grained feature processing across multiple dimensions.

Figure 3 illustrates a multi-branch convolutional module architecture designed to enhance the network’s feature extraction capabilities through Deep Separable Convolution (DSConv) and feature fusion operations. For the input, it is first divided into five groups along the channel dimension:

(4) $$X_{1},X_{2},X_{3},X_{4},X_{\mathrm{id}}=\mathrm{Split}\left(X\right)=X_{:g},X_{g:2g},X_{2g:3g},X_{3g:4g},X_{4g:},$$

where Split(·) denotes grouping and splitting along the channel dimension, *g* represents the number of channels in each group, $X_{:g}$ indicates taking the first *g* channels of X, $X_{g:2g}$ indicates taking the second *g* channels of X, and so on. Then, these five features are processed through five distinct operators:

(5) $$\begin{array}{cc} X_{1}^{\prime } & ={\mathrm{DSConv}}_{3\times 3\times 3}^{g\to g}\left(X_{1}\right), \end{array}$$

(6) $$\begin{array}{cc} X_{2}^{\prime } & ={\mathrm{DSConv}}_{1\times 1\times 11}^{g\to g}\left(X_{2}\right), \end{array}$$

(7) $$\begin{array}{cc} X_{3}^{\prime } & ={\mathrm{DSConv}}_{1\times 11\times 1}^{g\to g}\left(X_{3}\right), \end{array}$$

(8) $$\begin{array}{cc} X_{4}^{\prime } & ={\mathrm{DSConv}}_{11\times 1\times 1}^{g\to g}\left(X_{4}\right), \end{array}$$

(9) $$\begin{array}{cc} X_{\mathrm{id}}^{\prime } & =X_{\mathrm{id}}, \end{array}$$

where ${\mathrm{DSConv}}_{k_{1}\times k_{2}\times k_{3}}^{g\to g}$ denotes a separable convolution with depth, where both input and output channels are of size *g* and the kernel size is $k_{1}\times k_{2}\times k_{3}$, and $X_{\mathrm{id}}^{\prime }=X_{\mathrm{id}}$ denotes the “identity mapping”. Finally, the outputs from each branch are concatenated:

(10) $$X^{\prime }=\mathrm{Concat}\;(X_{1}^{\prime },\;X_{2}^{\prime },\;X_{3}^{\prime },\;X_{4}^{\prime },\;X_{\mathrm{id}}^{\prime }).$$

Our proposed InceptionDSConv3d module utilizes a multi-branch architecture combined with separable convolutional kernels of varying sizes to enable fine-grained feature processing across multiple dimensions. Simultaneously, deep separable convolutions substantially reduce parameter count and computational complexity, making the decoder more efficient while maintaining performance. This design is particularly well suited for restoring minute pores and complex boundaries in rock images. By capturing multi-scale contextual information and fusing features from different directions, it significantly enhances the decoder’s ability to reconstruct spatial details.

### 3.4. The BasicResDWSBlock Module

**Figure 4:**
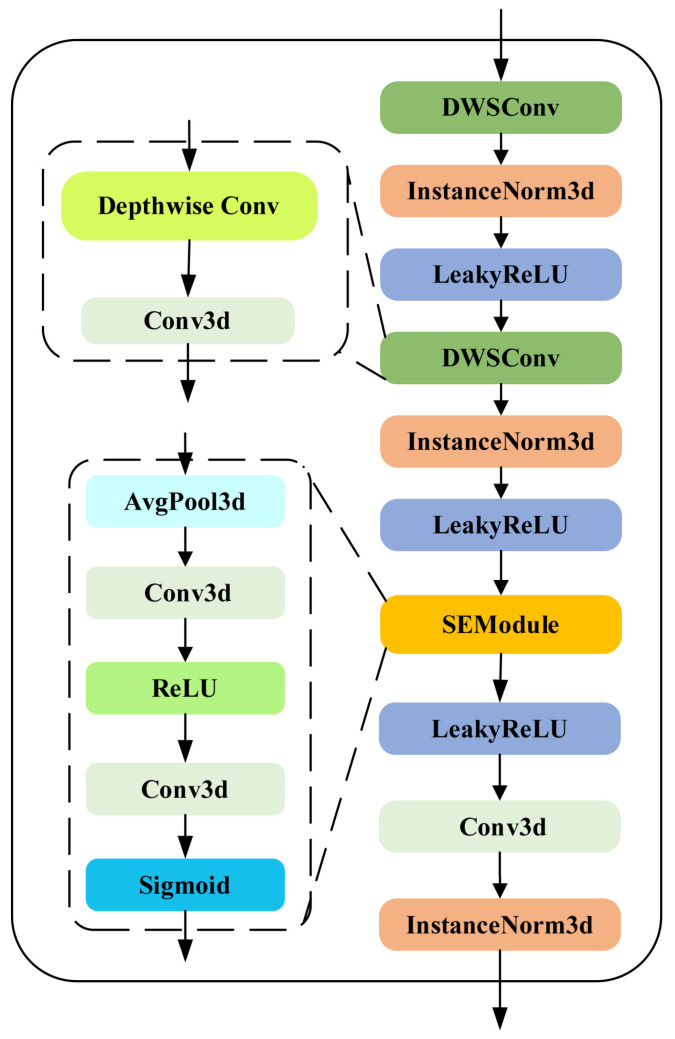
The BasicResDWSBlock module. This module adopts depthwise separable convolutions instead of standard convolutions and incorporates a separable activation layer after the second separable convolution block, achieving a significant reduction in model parameters and FLOPs.

Building upon the above work, to significantly reduce the number of parameters and FLOPs in the model, we propose the BasicResDWSBlock module by drawing inspiration from the Deep Separable Convolution approach [27]. Figure 4 depicts the BasicResDWSBlock module’s structure. In this module, depth-separable convolution is used in place of regular convolution. In contrast to a standard convolution, a depth-separable convolution will first convolve each channel of the input feature map independently in spatial dimension before combining the channels linearly by pointwise convolution.

Assume that the convolution kernel’s size in 3D is $K_{x}\times K_{y}\times K_{z}$, the input feature map’s size is D×H×W, the number of input channels is $C_{in}$, and the number of output channels is $C_{out}$. Then, the parametric quantities $P_{conv}$ and FLOPs $F_{conv}$ of the ordinary 3D convolution are as in Equations (11) and (12),

(11) $$P_{conv}=C_{in}\times C_{out}\times K_{x}\times K_{y}\times K_{z},$$

(12) $$F_{conv}=D\times H\times W\times C_{in}\times C_{out}\times K_{x}\times K_{y}\times K_{z},$$

and the parametric quantities $P_{dws}$ and FLOPs $F_{dws}$ of the 3D depth-separable convolution are as in Equations (13) and (14),

(13) $$P_{dws}=C_{in}\times K_{x}\times K_{y}\times K_{z}+C_{in}\times C_{out},$$

(14) $$F_{dws}=D\times H\times W\times C_{in}\times K_{x}\times K_{y}\times K_{z}+D\times H\times W\times C_{in}\times C_{out},$$

comparison of the formulas allows visualization of the changes in the number of parameters and calculations, which not only drastically reduces the number of parameters and calculations, but also speeds up the training and reasoning process. We also refer to the method of Hu et al. [36] to include the SE attention mechanism after the second depth-separable convolutional block of the BasicResDWSBlock module, and determined under several experiments that the channel compression parameter of the SE module is set to 16, and the number of compressed channels, $C_{squeezed}$, is given by Equation (15),

(15) $$C_{\mathrm{squeezed}}=C_{\mathrm{in}}/16,$$

after operations such as compression and activation functions, the channels are then expanded back for weighting operations on the original feature map.

## 4. Experiments and Results

### 4.1. Data Acquisition and Labeling

**Figure 5:**
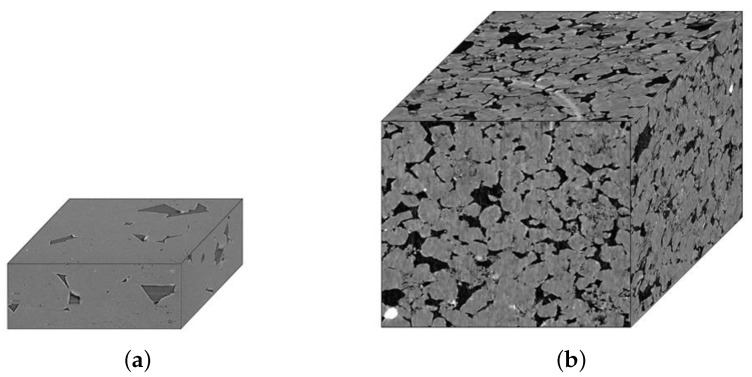
(**a**) µCT image of shale image. (**b**) µCT image of leopard sandstone image.

As shown in Figure 5, we utilized two datasets: one private dataset, Shale Images (dimensions 200 × 512 × 512), and another public dataset, Leopard Sandstone Images (dimensions 1000 × 1000 × 1000), sourced from the Digital Rock website [18].

During the annotation process of private data, the raw data is first sliced along a specific dimension to yield 200 two-dimensional images measuring 512 × 512 pixels. These images are then manually annotated using tools like LabelMe (v4.5.0) [37] and categorized into two classes: rock and pore. After annotation, a Python (v3.10.16) program converts multiple two-dimensional images into a three-dimensional format. Finally, the labels are saved in the .nii.gz format, suitable for deep learning training. In contrast, the raw and annotated data for the publicly available Leopard Sandstone Image dataset originate from the open-access Digital Rock website. Compared to this public dataset, our annotation process explicitly marks elongated pores and rigorously verifies annotation results. This distinction represents the core value of our dataset relative to the publicly available one.

**Figure 6:**
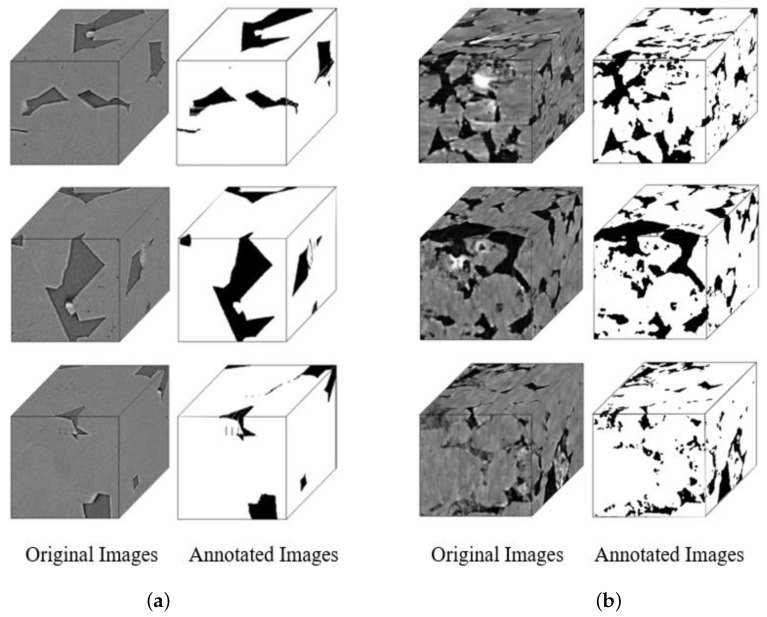
(**a**) shale images. (**b**) leopard sandstone images.

Due to the limited amount of data, we propose using a slicing method to create the dataset. After slicing the private dataset, Shale Images, using a Python program, we obtained 1024 image samples with dimensions of 200 × 200 × 200. The public Leopard Sandstone Images dataset was segmented using a Python program, yielding 216 image samples with dimensions of 250 × 250 × 250. Figure 6 illustrates selected segmented samples from both the Shale Images and Leopard Sandstone Images datasets.

As indicated in Table 1, we combine the two produced datasets into distinct resolutions and randomly separate them into training, validation, and test sets in a 4:1:1 ratio to guarantee that each set has rich pore distributions in order to increase the segmentation accuracy.

### 4.2. Experimental Setup

Our experimental environment configuration is shown in Table 2. The hyperparameter settings for the training phase include a batch size of 64 and a training cycle count of 500 epochs. Hyperparameters for the model training phase include an input image size of 200 × 200 × 200, an SGD optimizer (Meta Platforms, Inc., Menlo Park, CA, USA), a learning rate initialized at 0.001, and momentum decay and weight decay values set to 0.99 and 0.00003, respectively. During model training, a learning rate decay strategy was utilized, with all other hyperparameters set to the default values of U-Mamba-Bot.

### 4.3. Assessment of Indicators

In this study, we utilized the Dice similarity coefficient to evaluate the degree of overlap similarity between predicted and actual results, used IOU to measure the proportion of overlap between predicted and actual regions, and assessed the accuracy of the model’s predictions using the Accuracy metric [38]. To evaluate the scale and computational load of each model, we expressed the total number of floating-point operations (FLOPs) as the ratio of all floating-point operations to the time required to complete the task. In addition to the above performance metrics, we also calculated the number of network parameters (Params) and the inference time (Time) of the model on the test set.

### 4.4. Comparison of Different Segmentation Networks

We chose seven networks as comparison networks to assess the efficacy of our proposed approach. These include the most recent Mamba-based segmentation networks (U-Mamba-Bot and U-Mamba-Enc) and CNN-based techniques (3D U-Net, 3D SegNet, 3D U-ResNet, 3D KiUNet, and nnUNet). We train the aforementioned networks using the above two datasets in the same server environment, equipped with an NVIDIA GeForce RTX 4090 24 G GPU. Following training, performance metrics like accuracy, precision accuracy, and Dice similarity coefficient are computed to assess how well the model separates the segmented area from the background. For precise assessment of pore-scale properties [39], including fluid saturation, pore connectivity, and porosity, accurate segmentation is essential. We carefully designed the experimental methodology to periodically evaluate the performance of the model during training and saved checkpoints at the point of minimum validation loss to ensure that the most efficient version of the model was retained for subsequent use or further improvement. Table 3 shows the results of the comparison experiments using the dataset Shale Images, and Table 4 shows the results of the comparison experiments using the dataset Leopard Sandstone Images.

In comparison to 3D U-Net, 3D SegNet, 3D U-ResNet, 3D KiUNet, nnUNet, U-Mamba-Enc, and U-Mamba-Bot, our proposed approach better balances computational cost and performance among the benchmarks and produces excellent results with the same hyperparameters. While the amount of parameters, FLOPs, and inference time are relatively low, 3D U-Net, 3D SegNet, 3D U-ResNet, and 3D KiUNet exhibit poor segmentation accuracy and precision. In terms of segmentation accuracy and precision, UNet, U-Mamba-Enc, and U-Mamba-Bot outperform models like 3D U-Net; nevertheless, the model complexity is comparatively large. On the other hand, our enhanced model’s parameters, FLOPs, and inference time are, respectively, 66.83%, 56.19% and 50.41% lower than those of the baseline. In addition to being smaller than the baseline, our new model also has better accuracy, intersection ratio, and similarity coefficient. The comparison results show that for the pore segmentation task of 3D rock images, the method proposed in this paper can more efficiently balance the model’s lightweight characteristics and segmentation accuracy, which fully demonstrates the model’s suitability for real-time environments or resource-constrained environments.

**Figure 7:**
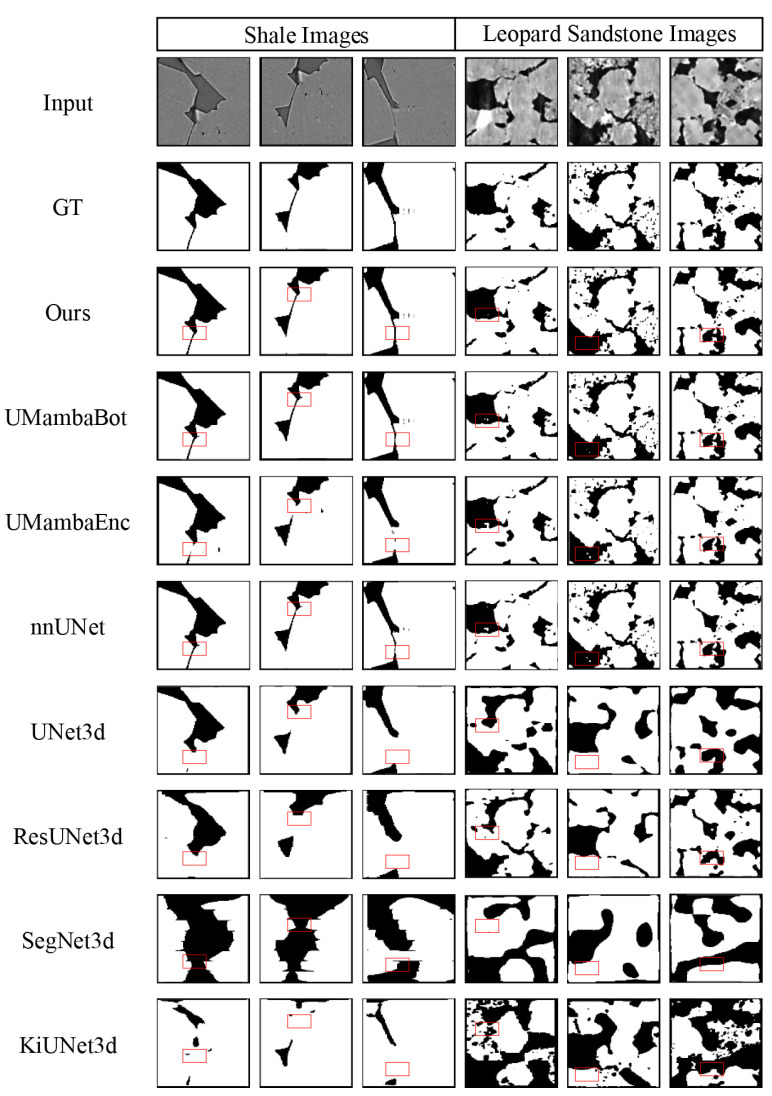
Qualitative comparison between LDLK-U-Mamba, U-Mamba-Bot, U-Mamba-Enc, nnUNet, 3D U-Net, 3D U-ResNet, 3D SegNet, and 3D KiUNet. Additionally, LDLK-U-Mamba shows better segmentation quality than others in terms of the segmentation of elongated pores and the integrity of the pore structure.

Figure 7 shows some of the segmentation results of the above models on the Shale Images test set and the Leopard Sandstone Images test set, and the visualization of the segmentation results shows that our proposed network not only accurately extracts the narrow and curved throat portion and extracts a more complete pore structure, but our proposed method also has stronger robustness and has fewer segmentation outliers.

### 4.5. Ablation Experiments

We created five sets of ablation experiments to compare the impact of the different improvement techniques on the model performance for two datasets, Shale Images and Leopard Sandstone Images, in order to assess the efficacy of the enhanced algorithms and demonstrate the model’s capacity for generalization. To guarantee comparable findings, we maintained the other hyperparameters at their default settings and utilized the same tools and datasets for the studies. The results of the ablation experiments using the Shale Images dataset are displayed in Table 5, and the results of the ablation experiments using the Leopard Sandstone Images dataset are displayed in Table 6. A indicates the use of the BasicResDWSBlock module, B indicates the use of the LDLK module, and C indicates the use of the InceptionDSConv3d module. The first row uses U-Mamba-Bot as the baseline, and each module can be added to the model separately.

To start, the LDLK module is used in the first three stages of the baseline encoder after taking into account Dice, accuracy, IOU, the number of parameters, and FLOPs. This module focuses on capturing global contextual information with dynamic large kernels to better model long-range 3D pore structures, so the ablation experiments’ results indicate that while the model’s number of parameters and FLOPs is slightly increased, Dice, accuracy, and IOU are improved. The InceptionDSConv3d module performs multi-scale feature fusion and refinement to accurately restore fine-grained pore boundaries, which is then used in the final two stages of the baseline decoder in order to improve the Dice, accuracy, and IOU metrics with the least amount of computational cost. The ablation experiments’ results indicate that the model’s number of parameters and FLOPs are slightly higher than the baseline, with an increase in Dice, accuracy, and IOU. Building upon the above work, to significantly reduce the number of parameters and FLOPs in the model, we propose the BasicResDWSBlock module. Ablation experiments demonstrate that BasicResDWSBlock reduces parameters and floating-point operations while maintaining accuracy through deep separable convolutions and SE attention mechanisms. Ultimately, we integrate all the enhancements to attain the best equilibrium outcomes. In comparison to the baseline, the LDLK-U-Mamba model’s parameters and FLOPs are reduced by 28.09 M and 547.27 GFLOPs, respectively, while accuracy, Dice, and IOU are improved by 0.12%, 1.25%, and 2.35%, respectively, according to the results of the ablation experiments conducted using the Shale Images dataset. In comparison to the baseline, the LDLK-U-Mamba model’s parameters and FLOPs decreased by 28.15 M and 547.27 GFLOPs, respectively, while accuracy, Dice, and IOU improved by 1.18%, 0.73%, and 1.39%, respectively, according to the results of the ablation experiments conducted with the dataset Leopard Sandstone Images.

## 5. Conclusions

In this study, we propose a lightweight 3D rock image pore segmentation method based on the Mamba architecture, termed the LDLK-U-Mamba model. To enhance segmentation performance, we propose the LDLK module during the encoding phase to capture global contextual information, and utilize the InceptionDSConv3d module during decoding to fuse and refine multi-scale features, yielding more accurate segmentation results. We also propose the BasicResDWSBlock module, utilizing deep separable convolutions and the SE module to reduce model parameters and computational complexity.

To evaluate the model’s performance, we tested segmentation accuracy, Dice score, intersection-over-union ratio, parameter count, FLOPs, and inference time. Segmentation results on the Leopard Sandstone Images dataset achieved a 99.38% accuracy, a 99.62% Dice score, and a 99.25% intersection-over-union ratio. The parameter count decreased from 42.12 M to 13.97 M, and FLOPs decreased from 973.91 G to 426.64 G. Experimental results demonstrate that the LDLK-U-Mamba model outperforms current mainstream segmentation methods across multiple key metrics.

Though our method demonstrates superior performance in 3D porous rock segmentation, it is difficult to generalize to other 3D segmentation tasks in different domains due to the inevitable domain differences. To address this, we plan to utilize transfer learning and fine-tuning strategies to optimize the model to enhance its generalizability for achieving 3D segmentation tasks in other domains.

## Figures and Tables

**Table 1:** Dataset division.

Dataset	Resolution	Train	Validation	Test	Total
Shale Images	200 × 120 × 120	683	171	170	1024
Leopard Sandstone Images	250 × 250 × 250	144	36	36	216

**Table 2:** Configuration of the experimental environment.

Category	Configuration
GPU	NVIDIA GeForce RTX 4090 24 G
System environment	Ubuntu 20.04
Torch version	2.6.0 + cu118
Programming language	Python 3.10.16

**Table 3:** Comparative experiment results utilizing the shale images dataset. **Bold** represents the best results, and underline represents the second-best results.

Method	Accuracy (%) ↑	Dice (%) ↑	IOU (%) ↑	Params (M) ↓	FLOPs (G) ↓	Time (ms) ↓
3D U-Net [6]	98.13	82.74	70.74	2.32	117.98	82.5
3D U-ResNet [24]	94.84	35.12	21.37	9.49	1699.84	1180.3
3D SegNet [12]	73.40	26.10	15.08	2.33	116.52	76.8
3D KiUNet [13]	94.57	55.90	39.09	2.33	130.17	89.2
nnUNet [14]	99.32	93.32	87.51	31.19	534.03	365.7
U-Mamba-Enc [17]	98.79	87.58	78.07	42.75	990.65	620.4
U-Mamba-Bot [17]	99.34	93.50	87.84	42.12	973.91	598.1
**Ours**	**99.46**	**94.67**	**89.90**	**14.03**	**426.64**	**295.3**

**Table 4:** Comparative experiment results utilizing the leopard sandstone images dataset. **Bold** represents the best results, and underline represents the second-best results.

Method	Accuracy (%) ↑	Dice (%) ↑	IOU (%) ↑	Params (M) ↓	FLOPs (G) ↓	Time (ms) ↓
3D U-Net [6]	88.77	92.90	86.74	2.32	117.98	83.2
3D U-ResNet [24]	86.32	91.36	84.10	9.49	1660.00	1165.8
3D SegNet [12]	65.44	75.55	60.74	2.33	116.54	77.4
3D KiUNet [13]	57.72	67.08	50.54	2.33	178.73	91.6
nnUNet [14]	98.04	98.80	97.66	31.19	534.03	368.2
U-Mamba-Enc [17]	81.13	89.55	81.13	42.75	990.65	625.1
U-Mamba-Bot [17]	98.22	98.90	97.89	42.12	973.91	602.5
**Ours**	**99.38**	**99.62**	**99.25**	**13.97**	**426.64**	**298.8**

**Table 5:** Results of the ablation experiments utilizing the dataset, Shale Images. **Bold** represents the best results.

A	B	C	Accuracy (%) ↑	Dice (%) ↑	IOU (%) ↑	Params (M) ↓	FLOPs (G) ↓
			99.34	93.50	87.84	42.12	973.91
✓			99.32	93.28	87.45	13.96	425.28
	✓		99.43	94.39	89.40	42.12	975.00
		✓	99.42	94.25	89.16	42.12	974.18
✓	✓	✓	**99.46**	**94.67**	**89.90**	**14.03**	**426.64**

**Table 6:** Results of the ablation experiments utilizing the dataset, Leopard Sandstone Images. **Bold** represents the best results.

A	B	C	Accuracy (%) ↑	Dice (%) ↑	IOU (%) ↑	Params (M) ↓	FLOPs (G) ↓
			98.22	98.90	97.89	42.12	973.91
✓			97.27	98.31	96.76	13.96	425.28
	✓		99.37	99.61	99.23	42.12	974.78
		✓	99.34	99.60	99.20	42.12	974.18
✓	✓	✓	**99.38**	**99.62**	**99.25**	**13.97**	**426.64**

## Data Availability

The original contributions presented in the study are included in the article. Further inquiries can be directed at the corresponding author.
